# Validation and Application of the Positive Play Scale Adapted for Chinese Gamblers: Its Relation to Disordered Gambling and Gambling Attitudes

**DOI:** 10.3389/fpsyg.2020.00263

**Published:** 2020-02-25

**Authors:** Kwok Kit Tong, Juliet Honglei Chen, Anise M. S. Wu

**Affiliations:** ^1^Department of Psychology, Faculty of Social Sciences, University of Macau, Macau, China; ^2^Centre for Cognitive and Brain Sciences, University of Macau, Macau, China

**Keywords:** responsible gambling, positive play, scale validation, gambling attitudes, Chinese community, gambling disorder

## Abstract

The Positive Play Scale (PPS) was designed to track the effectiveness of responsible gambling (RG) policy, with a focus on positive changes in beliefs and behaviors instead of the absence of problem gambling symptoms. The current study aimed to (1) validate the Positive Play Scale Adapted for Chinese gamblers (PPS-AC) using a probability Chinese community sample in Macao, (2) explore the relationships between the PPS-AC and symptoms of gambling disorder (GD), and (3) evaluate the associations of gambling attitudes with the PPS-AC. Through a two-step random sampling procedure, we interviewed 1,002 locally dwelling Macao Chinese adults (44.3% males; *M*_*age*_ = 44.28 years, *SD*_*age*_ = 17.35 years) by phone, in which 237 were past-year gamblers (49.8% males; *M*_*age*_ = 40.76 years, *SD*_*age*_ = 15.78 years). Results showed that a two-dimension structure fitted the data well for both the positive play behaviors scale and the positive play beliefs scale of the PPS-AC among past-year gamblers. In addition to findings of satisfactory internal consistency, the convergent validity of the PPS-AC was supported by its significant association with RG self-efficacy. All four PPS-AC constructs were negatively correlated with GD symptoms, whereas two behavior constructs of the PPS-AC significantly explained the variance of GD symptoms with negative valences. All gambling attitude dimensions were associated with at least one PPS-AC construct. The current study was the first to adapt the PPS on a probability Chinese community sample and extended its applicability. The findings support the PPS-AC as a reliable and valid tool for assessing positive play, which was negatively associated with symptoms of disordered gambling. Further, the significant associations between gambling attitudes and the PPS-AC provide insights for RG policies.

## Introduction

Responsible gambling (RG) was introduced to Macao in 2009, the only city in China where casino gambling is legal, with an objective to confine “gambling-related damage to a socially acceptable level” (Gaming Inspection and Coordination Bureau Macau, n.d.). This objective is consistent with Blaszczynski et al. (2004) statement that the role of RG policies is to minimize gambling-related harm. Even though some local stakeholders in Sydney have cast doubt on the effectiveness of RG in minimizing harm (Hing, 2003), RG practices have been found to be negatively related to gambling disorder (GD) symptoms in Chinese communities (Tong et al., 2018). One of the means to evaluate the effectiveness of RG policy is by considering the rate to which individuals are aware of RG policy. In Macao, surveys have found that the RG-awareness rate was 56.1–63.7% in the general population and 70.2–74.1% among gamblers (Institute for the Study of Commercial Gaming, 2017; Tong et al., 2019). In Macao, RG-aware people are likely to be better educated, have full-time and casino employment, and are also more likely to gamble responsibly (Tong et al., 2019). Nevertheless, Tong et al. (2019) also observed that RG-aware gamblers do not tend to report a high prevalence of adherence to a variety of RG practices (e.g. setting time limits, recording gambling details, seeking help if needed, signing up for self-exclusion if needed, not carrying a bank card when gambling, learning the risk of a game). Hence, there is a strong need for empirical research to find alternative ways to increase RG-awareness and RG practices in addition to common approaches (Blaszczynski et al., 2004).

In a focus group study on the relationship between RG practice and RG awareness, Chinese respondents were found to define RG narrowly as gamblers’ attempt to reduce or avoid negative outcomes (e.g. illegal acts and borrowing money), and they failed to recognize RG as a concerted effort by different stakeholders (Tong et al., 2018). The authors argued that RG was mistakenly considered to be more relevant to problem gamblers than to all gamblers and other stakeholders, which are viewpoints that render RG less effective in preventing GD. If RG is widely considered to be only relevant to problem gamblers, there may be a negative stigma against problem gamblers who are associated with RG, which may reduce adherence to RG practices and the promotion of RG among general gamblers, especially non-problematic ones.

In fact, Wood et al. (2018) also considered that the term RG may carry a negative connotation by specifying what a person should not do, and may reduce gamblers’ engagement in RG. To address this issue, Wood and Griffiths (2015) coined the term positive play, which refers to strategies that ordinary gamblers use to maintain a healthy gambling engagement level. In their study, they showed that positive players focused more on playing for recreation or leisure rather than attempting to alter their mood. They argued that gamblers would identify with the term positive play more than RG because they considered the latter more relevant to disordered gamblers. Instead of addressing the idea of gambling as having merely negative ramifications to the player, the emphasis on playing positively is suggested to be as important.

Based on information from a review of the literature, gamblers, and RG experts, Wood et al. (2017) developed the Positive Play Scale (PPS) to measure “healthy” gambling behaviors (c.f., problematic gambling behaviors). The PPS consists of two domains, namely, the PPS behaviors scale and the PPS beliefs scale. The positive play behaviors scale measures positive gambling behaviors with two components: honesty and control, and pre-commitment. The positive play beliefs scale, assessed by components of personal responsibility and gambling literacy, measures positive beliefs about gambling. The PPS was designed to evaluate the full spectrum of gamblers instead of focusing on only those at risk or disordered gamblers. With reference to Wood et al. (2018), honesty and control refers to players’ level of honesty about, and their control over, their gambling behaviors; pre-commitment refers to setting limits on time and money spent on gambling; personal responsibility refers to individuals’ beliefs that they are responsible for their gambling behaviors; and gambling literacy refers to beliefs regarding the nature of gambling.

The positive frame of the PPS may facilitate the promotion of RG behaviors among all gamblers, with and without GD. Moreover, the PPS can be used to evaluate the effectiveness of RG interventions because it is more sensitive to changes in non-disordered gamblers (Wood et al., 2018). Particularly in Macao, there is no reliable way to quantify the effectiveness of RG strategies, and most studies on RG policies have focused on RG awareness only (e.g. Institute for the Study of Commercial Gaming, 2017; Tong et al., 2018). The PPS may be used to evaluate the effectiveness of RG policies implemented by the government and gambling operators.

As the PPS has only been tested on conveniently sampled gamblers with disproportionately more senior gamblers (i.e., 40.6% in Study 2 and 58.4% in Study 3; Wood et al., 2017), a probability sample through random sampling method may enhance the generalization of results to more general gambler populations. The PPS could be particularly useful in Macao, which has the highest gross gambling revenue worldwide and a government that plays a prominent role in RG policies targeting the general public. A study using a randomly sampled community sample can better inform RG stakeholders on RG promotion strategies targeting the general public and general gamblers. Wood et al. (2018) have only demonstrated that the PPS is a valid measurement across Canada in a project commissioned by the Canadian Responsible Gambling Association, and hence its applicability to other cultures, such as the Chinese society of Macao, remains unknown. Furthermore, Wu and Lau (2015) have argued that there is a shortage of evaluative research on GD prevention services in Chinese populations. The PPS can be a potentially useful tool to evaluate the effectiveness of RG policies in Chinese societies. Thus, the first objective of the current study was to validate an adapted version of PPS for Chinese gamblers, the Positive Play Scale Adapted for Chinese gamblers (PPS-AC), with a probability Chinese community sample.

Given that the major goal of RG policies is to minimize harm resulting from gambling, it is desirable to know whether the PPS also serves as an indicator of lower levels of harm, such as a lower susceptibility to GD. Wood et al.’s (2017) findings showed a negative relationship between problem gambling (assessed by the Problem Gambling Severity Index, PGSI) and the PPS. Although certain PGSI items overlap with the symptoms of GD proposed by the DSM-5 (American Psychiatric Association [APA], 2013), the PGSI was specifically developed for community samples (Ferris and Wynne, 2001), which may omit some disordered gambling features that are important to clinical diagnoses. Practically, the DSM-5’s GD symptom list has been used to measure the severity of gambling problems and to inform policy (e.g. in Macao by the government and non-governmental organizations). It is warranted to examine the missing link between the DSM-5 GD symptoms and the PPS, which may promote its applicability in harm minimization in regions with large-scaled gambling industries like Macao and Singapore. Therefore, the second objective of the current study was to fill this knowledge gap and test whether the PPS has a negative association with DSM-5 GD symptoms. Some studies reported that gender and age were significant correlates of GD symptoms (e.g. Desai et al., 2007; Tang and Wu, 2012; Chen et al., 2018), whereas Wood et al. (2018) found that age was associated with the PPS. We thus further controlled for the effect of gender and age when exploring the association between the PPS-AC and GD symptoms.

Although beliefs and behaviors regarding positive play are potentially useful to evaluate the effectiveness of RG strategies, no study has evaluated the potential antecedents of positive play. We anticipated that gambling attitudes, as measured by the Inventory of Gambling Motives, Attitudes and Behaviors-Revised (GMAB-R; Tao et al., 2011; Wu et al., 2012), would be a promising candidate correlated with positive play. The GMAB-R attitudes domain taps into gambling attitudes in non- or sub-clinical gamblers by providing multiple perspectives for evaluating gambling engagement (Wu et al., 2012). The GMAB-R measures the subjective appraisals of gambling behaviors with four subscales, namely: negative consequences of gambling (e.g. family problems caused by gambling behaviors; hereinafter negative consequences), techniques (that influence winning or losing), superstition (i.e., believing that superstitious practices can increase the chance of winning in gambling) and fate and luck (that influences gambling outcomes). The relationship between attitudes and behaviors has not only been justified by theories (e.g. Fishbein and Ajzen, 2011) but also empirically supported by past studies. For example, GMAB-R attitudes were found to be significantly associated with non- and sub-clinical gambling behaviors (Tao et al., 2011; Wu et al., 2012), as well as gambling urges (Wu et al., 2013; Wang et al., 2016).

As for specific hypothesized relationships between the four GMAB-R attitude dimensions and positive play, a negative correlation between superstition and positive play was hypothesized because superstition has a positive correlation with gambling expense and frequency across different Chinese samples (Tao et al., 2011; Wu et al., 2012; Chen et al., 2015). Negative consequences have been found to be negatively associated with gambling urge, while technique and fate and luck have been found to be positively correlated with gambling urge (Wu et al., 2012). We, therefore, hypothesized salient relationships between the remaining three GMAB-R attitude dimensions and positive play, namely: positively valenced negative consequences, negatively valenced technique, and negatively valenced fate and luck. Because a significant association of GMAB-R attitudes with gender and age was found in some previous studies (e.g. Tao et al., 2011; Wu et al., 2012), as well as a significant age difference of positive play (Wood et al., 2018), we further controlled for gender and age in exploring to which extent the four GMAB-R attitude dimensions could explain the variance of positive play.

In sum, the present study aimed to (1) evaluate the psychometric properties of the PPS-AC in a probability Chinese community sample, (2) examine the PPS-AC’s relation to GD symptoms, and (3) test the PPS-AC’s relation to a validated measurement of Chinese gambling attitudes.

## Materials and Methods

### Participants

One thousand and two respondents, 44.3% males (95% CI [39.9%, 46.1%]) and 55.7% females (95% CI [52.6%, 58.8%]), took part in the study; their ages ranged from 18 to 89 years (*M* = 44.28 years, *SD* = 17.35 years) with 45.7% (*n* = 448) having attained a tertiary education or above. In this general sample (*N* = 1002), 456 respondents (46.4%) were self-identified as lifetime gamblers, in which 51.4% (*n* = 237) were past-year gamblers. The original PPS was developed for recent gamblers who have gambled in the past month. In order to make a meaningful exploration of the PPS-AC in relation to past-year GD symptoms and GMAB-R attitudes, the present investigation extended the use of the PPS-AC to gamblers who have gambled in the past year. Therefore, the validation and application of the PPS-AC were conducted with a past-year gambler sample (*N* = 237; age: range = 18–73 years, *M* = 40.76 years, *SD* = 15.78 years) of 49.8% males (95% CI [43.3%, 56.2%]) and 50.2% females (95% CI [43.8%, 56.6%]). In this past-year gambler sample, 0.8% of respondents (*n* = 2) attained no formal education/kindergarten, 9.3% (*n* = 22) attained a primary education, 14.3% (*n* = 34) a junior high school education, 19.0% (*n* = 45) a senior high school education, 11.4% (*n* = 27) a tertiary education subdegree, 44.3% (*n* = 105) had a tertiary education with a bachelor degree or above, and 0.8% (*n* = 2) did not specify their education. Regarding their job status, 63.3% (*n* = 150) of participants were full time employees, 3.4% (*n* = 8) worked part time, 3.8% (*n* = 9) were homemakers, 14.3% (*n* = 34) had retired, 14.3% (*n* = 34) were students, 5.9% (*n* = 14) were unemployed, and one did not report job status. Among the 158 employed participants, 24.1% (*n* = 38) worked in a casino and the rest (75.9%, *n* = 120) worked in other settings.

### Measures

#### Positive Play

Positive play was assessed by the PPS-AC, an adapted Chinese version of Wood et al.’s (2017) PPS. Similar to the PPS, the PPS-AC consists of two independently constructed domains: the two-construct positive play behaviors scale, and the two-construct positive play beliefs scale. For the positive play behaviors scale, the first construct of honesty and control was assessed by three items (e.g. I was honest with my family and/or friends about the amount of time I spent on gambling), while the second construct of pre-commitment was assessed by four items (e.g. I considered the amount of time I was willing to spend before I gambled). The positive play beliefs scale consists of two subscales: personal responsibility was assessed by four items (e.g. it is my responsibility to spend only money that I can afford to lose), while gambling literacy was assessed by three items (e.g. gambling is a good way to make money). In the Chinese version, all the PPS-AC items went through a formal English-to-Chinese translation and back-translation between English and Chinese by two professional translators. The translation accuracy and compatibility were further assessed by three bilingual psychologists. In order to compensate for the lack of visual aid during the telephone interview and to facilitate the response of local participants in the current study, we made adaptations to the original anchors of Wood et al.’s (2017) PPS items: the original seven-point Likert scale (1 = *strongly disagree* to 7 = *strongly disagree*) was revised as a five-point one (1 = *strongly disagree* to 5 = *strongly agree*) for positive play beliefs. A similar five-point Likert scale (1 = *strongly disagree* to 5 = *strongly agree*) was adopted for positive play behaviors with a modified opening guidance that “to what extent do you agree that each of the following statements precisely described your gambling behaviors in the past year,” which changed the original seven-point behavioral frequency Likert scale (1 = *never* to 7 = *always*) to one that assessed how positively one gambled in the past year.

#### Gambling Disorder Symptoms in the DSM-5

Consistent with previous telephone survey studies (e.g. Chen et al., 2018), the nine items from the DSM-5’s GD criteria were used on a dichotomous scale (1 = *yes*, 0 = *no*) to assess GD symptoms participants may have experienced over the past 12 months. A sample question is, “Have you experienced restlessness or irritability when attempting to cut down or stop gambling in the past 12 months?” The scale showed satisfactory reliability in the current study (KR-20 = 0.78).

#### Gambling Attitudes

Gambling attitudes were measured by the attitudes scale of the GMAB-R (Tao et al., 2011; Wu et al., 2012), which consists of four dimensions. Items are rated and scored on a five-point Likert scale (1 = *strongly disagree*, 5 = *strongly agree*). Negative gambling consequences were assessed by seven items (e.g. gambling drives one crazy; α = 0.70). Technique was assessed by six items (e.g. having great skills increases the chance of winning; α = 0.88). Superstition was assessed by four items (e.g. special numbers, colors, items, or clothes can increase your chances of winning; α = 0.70). The construct of fate and luck was assessed by three items (e.g. winning or losing depends on fate; α = 0.79). A subscale score was computed for each attitude dimension by averaging all the item scores within that subscale. A higher subscale score indicated a stronger gambling attitude regarding the corresponding dimension.

#### Responsible Gambling Self-Efficacy

RG self-efficacy was measured by a single-item on a five-point Likert scale, which measured respondents’ self-perception of their capability of practicing RG (e.g. To what extent do you think you are able to practice RG?). RG self-efficacy was included for testing the convergent validity of the PPS-AC because the PPS was designed to assess RG (Wood et al., 2018), and previous findings supported a positive association between generalized self-efficacy and some PPS constructs (Wood et al., 2017). Among past-year gamblers, only those who were aware of RG answered this question (*n* = 153).

#### Demographics

Demographic items included gender, age, education attainment (six categories: no formal education/kindergarten, primary, junior high, senior high, tertiary of sub-degree, tertiary of bachelor degree and above), and work status (six categories: full-time, part-time, homemaker, retired, student, unemployed), whereas employed participants were also asked whether they were casino staff (yes/no).

### Procedure

Our target participants were local residents aged 18 or above and able to speak Chinese (Cantonese or Mandarin). We approached participants by phone with a two-step random sampling procedure to acquire a probability community sample of 1,002 eligible participants. The first step of the random sampling picked up households’ numbers from the local telephone directory, and the second step entailed selecting an eligible participant from each household using the last-birthday rule. Participation was voluntary and did not involve monetary rewards. Following the guidelines and formula proposed by the American Association for Public Opinion Research (2016), the cooperation rate was calculated as 68.1%. Ethics approval was obtained from the affiliated university of the authors.

## Results

### Confirmatory Factor Analysis (CFA) of the PPS-AC

Because the positive play behavior scale and the positive play belief scale were developed separately for assessing two conceptually distinct constructs (i.e., behavior vs. belief; Wood et al., 2017), we followed the original conceptualization of PPS and evaluated the structure and dimensionality of a two-factor model of positive play behaviors and a two-factor model of positive play beliefs, respectively, with CFA by Mplus 7.4 (Muthén and Muthén, 1998–2012). The robust maximum likelihood (MLR) estimator was chosen with all the missing values taken into account and non-normality adjusted (Muthén and Muthén, 1998–2012; Muthén and Asparouhov, 2002). As recommended by Kline (2016), CFA models were assessed by the model chi-square test (χ^2^), with a non-significant χ^2^ indicating that the observed and implied variance-covariance matrices are similar. Additional three approximate fit indexes (RMSEA, CFI, and SRMR) were also reported for further references. A scale item is accepted when its factor loading is >0.30 (Hair et al., 2010).

After excluding one case that did not respond to the positive play behaviors scale (*N* = 236 after the removal), we tested this seven-item two-factor scale using CFA and found the model fit of the original structure, without any postulated covariance, not satisfactory, χ^2^(13) = 93.09, *p* < 0.001, RMSEA = 0.162, 90% CI [0.132,0.193], CFI = 0.779, SRMR = 0.152. We further examined the residual covariance matrix for sources of misfit and added two residual covariances to modify the model: one between Item-HC2 (i.e., honest with my family and/or friends about the amount of money spent on gambling) and Item-HC3 (i.e., honest with my family and/or friends about the amount of time spent on gambling), and another between Item-PC3 (i.e., only gambled with money that I could afford to lose) and Item-PC4 (i.e., only spent time gambling that I could afford to spend). The modified model displayed a satisfactory model fit, with χ^2^(11) = 12.77, *p* = 0.31, RMSEA = 0.026, 90% CI [0.000, 0.076], CFI = 0.995, SRMR = 0.055, and the standardized factor loadings of all the items were >0.30 (Table 1). This model was also better than its one-factor unconstrained model, with χ^2^(14) = 183.62, *p* < 0.001, RMSEA = 0.227, 90% CI [0.198, 0.256], CFI = 0.531, SRMR = 0.130. Therefore, this model was chosen as the final positive play behaviors measurement model.

**TABLE 1:** Confirmatory factor analysis results of positive play behavior items among past-year gamblers (*N* = 236).

**PPS-AC positive play behavior items**	**Standardized factor loadings**
**Honesty and control (HC)**	
HC1: I felt in control of my gambling behavior	0.69***
HC2: I was honest with my family and/or friends about the amount of money I spent gambling	0.64***
HC3: I was honest with my family and/or friends about the amount of time I spent gambling	0.61***
*HC2 with HC3*	0.77***
**Pre-commitment (PC)**	
PC1: I considered the amount of money I was willing to lose before I gambled	0.79***
PC2: I considered the amount of times I was willing to spend before I gambled	0.92***
PC3: I only gambled with money that I could afford to lose	0.46***
PC4: I only spent time gambling that I could afford to spend	0.50***
*PC3 with PC4*	0.77***
**PC with HC**	0.90***

***p < 0.001. The Italicized value denotes residual covariance.

Regarding the seven-item positive play beliefs scale (*N* = 237), the CFA result showed that the original two-factor structure, without any postulated covariance, fit the data well, with χ^2^ (13) = 16.15, *p* = 0.24, RMSEA = 0.032, 90% CI [0.000.076], CFI = 0.983, SRMR = 0.050, and the standardized factor loadings of all the items were >0.30 (Table 2). Compared to its one-factor unconstrained model, this two-factor model displayed model-fit superiority, with its counterpart of χ^2^(14) = 68.24, *p* < 0.001, RMSEA = 0.128, 90% CI [0.098, 0.159], CFI = 0.709, SRMR = 0.080. Therefore, this original two-factor model was chosen as the final positive play belief measurement model.

**TABLE 2:** Confirmatory factor analysis results of positive play belief items among past-year gamblers (*N* = 237).

**PPS-AC positive play belief items**	**Standardized factor loadings**
**Personal responsibility (PR)**	
PR1: I should be able to walk away from gambling at any time	0.66***
PR2: I should be aware of how much money I spend when I gamble	0.56***
PR3: It’s my responsibility to spend only money that I can afford to lose	0.75***
PR4: I should only gamble when I have enough money to cover all my bills first	0.64***
**Gambling literacy (GL)**	
GL1: Gambling is not a good way to make money	0.33***
GL2: If I gamble more often, it will help me to win more than I lose	−0.77***
GL3: My chances of winning get better after I have lost	−0.85***
**PR with GL**	0.53***

***p < 0.001.

The reliability of each subscale was computed using McDonald’s omega, which does not assume zero correlations between the PPS-AC item residuals and identical factor loadings for PPS-AC items on a given subscale. For comparison with existing studies on the PPS we computed Cronbach’s alpha, though it is not recommended to use Cronbach’s alpha in the present study because the assumptions of zero residual correlations and identical factor loadings were violated. Among past-year gamblers, good internal consistency was found in the subscales of honesty and control (McDonald’s ω = 0.84, Cronbach’s α = 0.82), and pre-commitment (McDonald’s ω = 0.81, Cronbach’s α = 0.81). In contrast, marginally acceptable internal consistency was found in the subscales of personal responsibility (McDonald’s ω = 0.76, Cronbach’s α = 0.75) and gambling literacy (McDonald’s ω = 0.70, Cronbach’s α = 0.65). Regarding the convergent validity, we found that honesty and control and pre-commitment were positively related to RG self-efficacy (*r* = 0.24 and 0.34, respectively) at significant levels (*p*s < 0.05). Positive play belief constructs, personal responsibility, and gambling literacy were also positively related to RG self-efficacy (*r* = 0.34 and 0.20, respectively) at significant levels (*p*s < 0.05).

### PPS-AC and GD Symptoms

The associations between the PPS-AC and GD symptoms were examined with bivariate correlations (Table 3). As expected, the two behavior constructs of the PPS-AC (honesty and control, and pre-commitment) were significantly and negatively related to GD symptoms (*r* = –0.35 and –0.40, respectively, *p*s < 0.001). Likewise, the two belief constructs of the PPS-AC (personal responsibility and gambling literacy) were significantly and negatively related to GD symptoms (*r* = –0.44 and –0.32, respectively, *p*s < 0.001).

**TABLE 3:** Bivariate correlations of PPS-AC constructs and other key variables.

	***N***	***M***	***SD***	**1**	**2**	**3**	**4**
1. Positive play behavior-HC	236	3.95	0.91	1	−	−	−
2. Positive play behavior-PC	236	4.16	0.72	0.63***	1	−	−
3. Positive play behavior-PR	236	4.15	0.65	0.55***	0.68***	1	−
4. Positive play behavior-GL	237	4.07	0.79	0.26***	0.32***	0.42***	1
5. RG Self-efficacy	153	4.20	0.95	0.24**	0.34***	0.34***	0.20*
6. GD symptoms	237	0.68	1.40	−0.35***	−0.40***	−0.44***	−0.32***
7. Negative consequences	236	3.81	0.54	0.03	0.08	0.19**	0.25***
8. Technique	235	2.67	0.96	–0.09	–0.003	–0.11	−0.43***
9. Superstition	236	2.36	0.85	−0.17**	−0.14*	−0.22**	−0.34***
10. Fate and luck	236	3.52	0.92	0.03	0.10	0.20**	0.11

HC, honesty and control, PC, pre-commitment; PR, personal responsibility; GL, gambling literacy; RG, responsible gambling; GD = gambling disorder. *p < 0.05, **p < 0.01, ***p < 0.001.

A hierarchical regression analysis was conducted to test whether the PPS-AC accounted for individual differences in GD symptoms after controlling for the effects of gender and age (Table 4). Results indicated that the PPS-AC accounted for additional variance beyond the effects of gender and age, Δ*F* (4, 225) = 14.95, *p* < 0.001. However, only the positive play belief constructs of personal responsibility and gambling literacy were significant predictors (β = –0.25 and –0.15, respectively, *p*s < 0.05).

**TABLE 4:** Hierarchical regression of PPS-AC predicting GD symptoms (*N* = 232).

	**GD symptoms (β)**
**Step 1**	
Gender	−0.20**
Age	0.13*
*F* (2, 229)	6.93**
*R*^2^	0.057
**Step 2**	
Gender	–0.09
Age	0.06
*Honesty and control*	–0.09
*Pre-commitment*	–0.10
*Personal responsibility*	−0.25**
*Gambling literacy*	−0.15*
Δ*F* (4, 225)	14.95***
Δ*R*^2^	0.198

*p < 0.05, **p < 0.01, ***p < 0.001.

### PPS-AC and GMAB-R

Bivariate correlations between the attitude subscales of the GMAB-R and the PPS-AC were conducted. Both behavior constructs of the PPS-AC (honesty and control, and pre-commitment) were significantly and negatively related to the GMAB-R superstition (*r* = –0.17 and –0.14, respectively, *p*s < 0.05). Personal responsibility of the positive play beliefs scale was significantly and negatively related to the GMAB-R superstition (*r* = –0.22, *p* < 0.05), and significantly and positively related to the GMAB-R negative consequences as well as fate and luck (*r* = 0.19 and 0.20, respectively, *p*s < 0.01). Gambling literacy of the positive play belief construct was significantly and negatively related to the GMAB-R superstition and technique (*r* = –0.34 and 0.43, respectively, *p*s < 0.001) and positively related to the GMAB-R negative consequences (*r* = 0.25, *p* < 0.001).

Four hierarchical regression analyses were conducted to test the extent to which gambling attitudes (GMAB-R attitudes) accounted for variance in the PPS-AC, after controlling for the effects of gender and age (Table 5). We performed listwise deletion to handle missing data on the variable-level, which excluded cases with one or more missing values of those involved variables. Results indicated that the GMAB-R accounted for additional variance beyond the effects of gender and age: pre-commitment [Δ*F*(4, 223) = 4.30, *p* < 0.01], personal responsibility (Δ*F*(2, 223) = 9.47, *p* < 0.001), and gambling literacy [Δ*F*(2, 224) = 19.44, *p* < 0.001]. In particular, superstition was a significant predictor of honesty and control, pre-commitment, personal responsibility, and gambling literacy with negative valences (β = –0.19, –0.24, –0.31, and –0.18, respectively). Negative consequence was a significant predictor of personal responsibility and gambling literacy with positive valences (β = 0.13 and 0.23, respectively). Technique was a significant predictor of gambling literacy with a negative valence (β = –0.32). Fate and luck was a significant predictor of pre-commitment and personal responsibility, with positive valences (β = 0.17 and 0.25, respectively).

**TABLE 5:** Hierarchical regressions of GMAB-R attitudes predicting PPS-AC constructs.

	**Positive play behaviors**		**Positive play beliefs**	
	**HC (*N* = 230)**	**PC (*N* = 230)**	**PR (*N* = 230)**	**GL (*N* = 231)**
**Step 1**				
Gender	0.25***	0.15**	0.19**	0.18**
Age	−0.15*	−0.26***	–0.12	–0.03
*F (df* = *2, 227/228)*	10.68***	11.40***	6.13**	3.80*
*R*^2^	0.086	0.091	0.051	0.032
**Step 2**				
Gender	0.25***	0.16*	0.18**	0.07
Age	−0.16*	−0.29***	−0.18**	−0.12*
*Negative consequences*	0.02	0.08	0.13*	0.23***
*Technique*	0.07	0.15	0.10	−0.32***
*Superstition*	−0.19*	−0.24**	−0.31***	−0.18*
*Fate and luck*	0.11	0.17*	0.25***	0.09
Δ*F* (*df* = *4, 223/224*)	1.96	4.30**	9.47***	19.44***
Δ*R*^2^	0.031	0.065	0.138	0.249

HC, *Honesty and control*; PC, *Pre-commitment*; PR, *Personal responsibility*; GL, *Gambling literacy*. Standardized coefficients are reported for each hierarchical regression. *p < 0.05, **p < 0.01, ***p < 0.001.

## Discussion

The present study is the first study to test the applicability of the PPS-AC in a probability Chinese community sample. A two-factor structure was found to be satisfactorily fitted for both positive play behaviors and positive play beliefs with adapted five-point Likert scales. It is worth noting that our adaptation to the anchors of the positive play behavior scale (i.e., from frequency to agreement level) only measure the general extent of one’s positive play behaviors, instead of the originally designed frequency of positive play behaviors, and hence precludes a direct comparison to Wood et al.’s (2017) findings on positive play behaviors. As such, future studies are also recommended to use the response scale of the original PPS when evaluating or designing related gambling policies and interventions. The adapted version of the PPS for Chinese gamblers, the PPS-AC, showed acceptable internal consistency and its significant, positive bivariate correlation with RG self-efficacy also demonstrated good convergent validity. While the original study employed a convenient gambler sample with disproportionately more senior gamblers (Wood et al., 2017), which undermined its applicability to other gamblers, the current study employed a random community sample, showing that the PPS-AC is a promising tool to indicate responsible and positive gambling behaviors and beliefs among general gambler populations. Hence, the PPS-AC can also serve as a tool to measure changes in positive play behaviors and beliefs in order to assess the effectiveness of RG policies or interventions targeting general gamblers. Currently, there is no study evaluating its utilities in interventions and further study is required to verify whether it is an effective tool. Although there are other measures related to RG, one of the advantages of the PPS-AC is that it is concise, reliable, and valid. It is a particularly useful tool in time-restricted situations, such as a telephone survey or an intercept survey. Tong et al. (2018) found that RG policies’ effectiveness was questioned by some stakeholders, including casino employees and gamblers, and the introduction of a tool for tracking positive play behaviors can help not only provide evidence of RG policies effectiveness but also enhance the perceived efficacy of stakeholders’ efforts to implement such policies.

Moreover, our findings suggest that the adapted PPS, the PPS-AC, can be used on the Chinese population. Wu and Lau (2015) argued that there may be a positive social norm in favor of gambling in Chinese culture, which may heighten the risk of GD among Chinese people. While RG is a collaborative effort between stakeholders in reducing harm (Blaszczynski et al., 2008), a tool that can reliably assess RG behaviors is essential for tracking the effectiveness of various RG intervention policies targeting general gamblers. Being the only city in China where casino gambling is legal, about half of the 16 million Chinese mainland visitors to Macao have visited casinos (Zeng et al., 2014). The government is one of the major stakeholders in implementing policies aimed at minimizing harm (Gaming Inspection and Coordination Bureau Macau, n.d.). RG awareness was employed to evaluate RG effectiveness (Tong et al., 2018), but its major limitation is its failure to indicate to what extent an RG policy can influence gamblers’ gambling behaviors. The PPS-AC can be used to evaluate policy effectiveness and provide unique information, in addition to measuring RG awareness rates and GD prevalence as suggested by previous studies (e.g. Wood et al., 2017, 2018).

This study also showed that the PPS-AC was negatively related to GD symptoms, which suggests that GD patterns may be restrained with more positive play behaviors. The practical advantage of using the PPS-AC over GD measures is that it is more sensitive to non- or sub-clinical gambling behaviors. Compared with using DSM-5’s GD symptom list (American Psychiatric Association [APA], 2013), which assesses prominent symptoms of the disorder, it may be easier and more suitable to administer the PPS-AC to trace the effectiveness of RG policies among general gamblers, with or without GD.

The GMAB-R attitude dimensions were found to be related to the PPS-AC constructs. In particular, positive play behaviors may be enhanced by a reduction in superstitious attitudes, which refers to the endorsement of superstitious ways to improve the chance of winning. Our results suggest that reducing this illusion of control may promote positive gambling behaviors and may restrain problematic gambling (Goodie, 2005). On the other hand, positive play beliefs may be enhanced by weakening favorable attitudes regarding superstition and techniques, as well as increasing endorsement of negative consequences related to gambling. Unexpectedly, fate and luck were positively associated with two PPS-AC constructs: pre-commitment and personal responsibility. However, it is premature to make any definitive conclusions on whether the effect of fate and luck is consistently different to other cognitive distortions (e.g. superstitions) on positive play, and thus further cross-examinations are required in future studies.

The relationship between the GMAB-R attitudes and the PPS-AC displayed an inverted pattern of that between the GMAB-R attitudes and GD, which may partially explain why GMAB-R attitudes were found to be related to GD (Wu et al., 2012; Chen et al., 2015). Consistent with attitude-behavior theories, our findings suggest that cognitive modification strategies may be employed to alter gambling attitudes and in turn to promote positive play beliefs and behaviors. For example, intervention strategies to improve positive play beliefs and behaviors can focus on lowering cognition distortions related to the role of superstition and techniques in gambling, as well as understanding the potentially detrimental consequences of gambling. Further studies are required to evaluate the causal relationship between the PPS-AC and gambling attitudes.

In conclusion, the current study is the first to adapt the PPS to a Chinese community, which is considered vulnerable to gambling problems by researchers (Wu and Lau, 2015). It has also filled the research gap regarding the PPS applicability and showed that the adapted PPS for Chinese gamblers, the PPS-AC, is a reliable and valid measure that can be applied to community-dwelling gamblers. Also, the PPS-AC is a concise and potentially useful tool in the sense that its scores negatively relate to GD symptoms. Finally, the current findings support that some gambling attitudes were associated with positive play beliefs and behaviors, and this provides practical insights for designing and implementing future RG strategies.

## Data Availability Statement

The datasets generated for this study are available on request to the corresponding author.

## Ethics Statement

The studies involving human participants were reviewed and approved by Panel on Research Ethics, University of Macau. The ethics committee waived the requirement of written informed consent for participation.

## Author Contributions

KT led the conceptualization and implementation of the study, including the literature search, analysis and interpretation of the data, and manuscript writing. JC contributed to the data collection, data analysis, data interpretation, and manuscript preparation. AW involved in the research conceptualization, data interpretation, and manuscript revision.

## Conflict of Interest

The authors declare that the research was conducted in the absence of any commercial or financial relationships that could be construed as a potential conflict of interest.
